# Methanol Oxidation Reaction in Alkaline Media Using Gold Nanoparticles Recovered from Electronic Waste

**DOI:** 10.3390/ma17061267

**Published:** 2024-03-09

**Authors:** Mariana Baruch-Soto, Lorena Magallón-Cacho, Jeannete Ramírez-Aparicio, Jesús Ortega-Guzmán, Edgar Borja-Arco

**Affiliations:** 1 Department of Theoretical Physics and Chemistry, Faculty of Chemistry, National Autonomous University of Mexico, Mexico City 04510, Mexico; 314311511@quimica.unam.mx; 2 CONAHCYT-Center for Research in Engineering and Applied Sciences, Autonomous University of the State of Morelos, Cuernavaca 62209, Mexico; lorena.magallon@conahcyt.mx (L.M.-C.); jramireza@conahcyt.mx (J.R.-A.); 3 Condensed Matter Department, Physics Institute, National Autonomous University of Mexico, Mexico City 04510, Mexico; iqortegag@gmail.com

**Keywords:** electronic waste, gold nanoparticles, electrocatalysis, methanol oxidation, alkaline fuel cells

## Abstract

This study investigates the potential of using gold nanoparticles (Au NPs) synthesized from e-waste as electrocatalysts for the methanol oxidation reaction (MOR), with the aim of applying them as an anode in alkaline direct methanol fuel cells (ADMFCs). The research addresses the pressing environmental challenge of e-waste disposal and explores the recycling of e-waste to obtain valuable materials for sustainable applications. Vulcan-supported gold nanoparticles (Au_e-w_/C NPs) are synthesized from gold coatings recovered from Intel Pentium 4 processor pins, demonstrating the feasibility of e-waste as electrocatalyst precursors. Comprehensive characterization techniques such as UV-Vis spectroscopy, high-resolution transmission and transmission electron microscopy (HR-TEM, TEM), selected area electron diffraction (SAED), scanning electron microscopy (SEM), and X-ray diffraction (XRD) are employed to evaluate the structural properties of the electrocatalyst. Electrochemical evaluation in 0.5 M KOH electrolyte by cyclic voltammetry reveals that the synthesized Au_e-w_/C NPs exhibit electrocatalytic activity (25.5 mA·mg^−1^_Au_) comparable to their commercially synthesized counterparts (30.1 mA·mg^−1^_Au_). This study highlights the potential for sustainable approaches in the production of electrocatalysts by utilizing e-waste as a source of valuable catalyst materials. It represents a pioneering effort in harnessing e-waste as a sustainable resource, offering new avenues for sustainable energy technologies while addressing environmental concerns and technological challenges in the field of ADMFCs.

## 1. Introduction

In today’s technology-driven world, electronic waste (e-waste) has become a pressing environmental issue, driven by widespread disposal of electronic devices and rapid technology obsolescence. This accumulation of discarded electronic components underscores the dual challenges of the need for sustainable disposal methods to mitigate environmental damage and the potential for resource recovery from e-waste [1,2,3,4,5]. Factors contributing to the increase in e-waste generation include inadequate waste management infrastructure, low awareness of responsible disposal practices, the tendency not to repair and reuse devices, rapid technological advances leading to obsolescence, and deficiencies in information systems for waste tracking and management. As a result, e-waste continues to accumulate at an alarming rate, posing significant environmental and health risks.

Recognizing the critical need for e-waste recycling is imperative to address these multifaceted challenges and encourage a more sustainable approach to technology waste management. Effective e-waste recycling initiatives have the potential to mitigate environmental impact, conserve resources, and reduce health risks associated with improper disposal [6,7,8,9]. In addition, e-waste recycling can stimulate economic growth by recovering valuable materials and creating opportunities in the recycling and circular economy sectors. Integrating e-waste recycling into broader sustainability efforts is vital to building resilient and environmentally conscious societies.

In addition, the chemical composition of e-waste varies depending on factors such as type, model, manufacturer, date of manufacture, and age of the electronic device [10]. For example, e-waste from computer and telecommunication systems often contains high levels of precious metals, such as copper, tin, silver, gold, and palladium [7]. This variability underscores the complexity of e-waste composition and presents challenges and opportunities for recycling and resource recovery efforts. The recognition of e-waste as a reservoir of valuable materials has sparked great interest in exploring its potential as a sustainable resource.

Gold, an essential component of many electronic devices, is an example of this valuable resource present in e-waste. Consequently, there is a great deal of research interest in the recovery of gold from e-waste to address environmental concerns and to take advantage of the resource. Several methods, from chemical leaching to more sustainable approaches, have been developed to extract and purify gold from electronic components [11,12,13,14]. Gold nanoparticles (Au NPs) have garnered attention in nanotechnology due to their unique properties and versatile applications in catalysis, biomedical applications, environmental remediation, and electrochemical applications [15,16,17,18,19,20,21,22].

On the other hand, the continued development of energy conversion technologies, such as ADMFCs, is crucial to meet the demand for clean and sustainable energy sources. ADMFCs offer promising avenues for efficient energy conversion, with the potential to use non-precious metal catalysts to reduce costs [23]. However, the performance of ADMFCs depends on the efficiency of the electrocatalysts, especially at the anode, where the fuel oxidation reaction takes place [24,25]. The demand for efficient electrocatalysts for ADMFCs [26,27] coincides with the search for cleaner and more sustainable energy solutions.

In this context, Au NPs have attracted attention for their remarkable electrocatalytic properties. This study delves into the electrocatalytic characteristics of Au NPs recovered from e-waste, exploring their suitability as electrocatalysts for the methanol oxidation reaction (MOR) in alkaline media. Harnessing Au NPs from e-waste as electrocatalysts underscores a sustainable approach aligned with recycling and resource efficiency. By demonstrating the feasibility and sustainability of e-waste recycling for electrocatalyst synthesis, particularly for MOR in alkaline media, this research underscores the potential of utilizing e-waste as a resource for efficient electrocatalysts. Ultimately, it offers a sustainable approach for ADMFCs’ applications, addressing environmental concerns and moving towards a circular economy [28,29,30].

## 2. Materials and Methods

### 2.1. Synthesis of Au/C NPs

The synthesis of Au_e-w_/C NPs comprised four distinct stages. Initially, gold coatings from Intel Pentium 4 processor pins were recovered using the methodology proposed by Su et al. [31], involving an acid digestion to selectively dissolve accompanying metals (Cu, Fe, Ni) present in the pins. Subsequently, HAuCl_4_ was synthesized from the recovered gold coatings. In this step, 2 mg of gold coatings were weighed and reacted with 5 mL of 10% *v/v* HCl (37%, Meyer, Mexico City, Mexico) and 2 μL of concentrated HNO_3_ (69%, Meyer, Mexico City, Mexico) in a conventional synthesis reactor (Monowave 50, Anton Paar, NSW, Australia), applying for this purpose a heating ramp up to 160 °C for 10 min; once this temperature was reached, it was maintained for an additional 2 min. After synthesis, HAuCl_4_ was recrystallized in a vacuum desiccator. After evaporating the solvent, HAuCl_4_ was transferred to a 10 mL volumetric flask and adjusted with deionized water to obtain a 1 mM concentration. The third part involved the synthesis of Au NPs from HAuCl_4_. For this, 5 mL of the prepared 1 mM HAuCl_4_ solution was reacted with 0.5 mL of 38.8 mM sodium citrate (C_6_H_5_Na_3_O_7_⋅2H_2_O, 99.0–100.1%, J.T. Baker, Quebec, QC, Canada) under conditions identical to those used for HAuCl_4_ synthesis. The synthesis process was duplicated, yielding a total of 11 mL of Au NPs solution. Finally, the obtained Au NPs were supported on Vulcan^®^ XC-72 (Cabot, Boston, MA, USA) via magnetic stirring for 24 h (Au_e-w_/C NPs). The supported Au NPs were vacuum-dried, resulting in a black powder, at a loading of ~20 wt% Au_e-w_/C NPs.

On the other hand, in order to compare the structural and electrocatalytic activity of Au_e-w_/C NPs obtained from e-waste recycling, Au_com_/C NPs were synthesized using a commercial precursor (HAuCl_4_, 99.99%, Sigma-Aldrich, St. Louis, MO, USA), at the same synthesis conditions.

In this way, this comprehensive synthesis protocol ensured the production of gold nanoparticles supported on Vulcan for subsequent structural and electrochemical characterization.

### 2.2. Structural Characterization of Au NPs and Au/C NPs

Unsupported Au NPs were characterized by UV-vis spectroscopy using a Genesys 150 spectrophotometer (ThermoFisher-SCIENTIFIC, Waltham, MA, USA) within the wavelength range of 250–1100 nm; TEM, HR-TEM, and SAED studies were performed using a JEM2010-FEG microscope (Jeol, Tokyo, Japan) at 200 kV. On the other hand, the supported Au NPs were also subjected to comprehensive characterization. SEM was performed using a JSM 7800F microscope (SEMTech Solutions, North Billerica, MA, USA) at 15 kV. Additionally, Energy Dispersive X-ray Spectroscopy (EDS) was conducted with an OXFORD spectrophotometer coupled to the JSM 7800F microscope. Further structural data were obtained by XRD using a Bruker D8 Discover diffractometer (Cu-Kα1 radiation, 1.54060 Å, Bruker Mexicana, Ciudad de México, Mexico). TEM and HR-TEM studies were also performed.

### 2.3. Electrochemical Characterization of Au/C NPs

Electrochemical studies were carried out using a three-electrode electrochemical cell. A saturated Calomel electrode was used as a reference electrode, a graphite rod as a counter electrode, and as a working electrode, an Au/C NP electrocatalytic ink deposited on a glassy carbon disk (Ø = 5.0 mm). The measured potential values are referred to as the reversible hydrogen electrode (RHE). The electrolyte used was a 0.5 M KOH solution (≥98%, Sigma-Aldrich, St. Louis, MO, USA). The electrocatalytic ink preparation process consisted of mixing 2 mg of the respective electrocatalyst (Au/C NPs), 298 µL of deionized water (18.2 MΩ·cm), 200 µL of isopropyl alcohol (99.5%, Meyer, Mexico City, Mexico), and 2 µL of Nafion (5% in aliphatic alcohols, Sigma-Aldrich, San Louis, MO, USA). The mixture was sonicated using a sonicator (Cole-Palmer, Vernon Hills, IL, USA) until the ink was homogeneously dispersed. Subsequently, 5 μL of the resulting ink was deposited on the glassy carbon disk electrode, previously polished using MicroCloth with 5 and 0.3 μm alumina abrasives. Electrochemical data were acquired using a bipotentiostat/galvanostat (PINE, Wavedriver, AFP2, Durham, NC, USA) and commanded by AfterMath^®^ software (v 1.6.10513, Durham, NC, USA) developed by the same company.

Cyclic voltammetry (CV) studies were performed in the absence of methanol in alkaline media to characterize the electrode surface (Au/C NPs ink). For this purpose, the electrolyte was previously purged with pure N_2_ (UHP, Praxair, Mexico City, Mexico) for 30 min. Potential sweeps were performed for 16 cycles, from 0.46–1.56 V/RHE at a 20 mV·s ^−1^ scan rate. For the electrochemical study of the methanol oxidation reaction, CV studies were performed in the presence of methanol at different concentrations (from 1–5 M) (99.8%, Sigma-Aldrich, St. Louis, MO, USA) in alkaline media, using the same conditions as in the absence of methanol.

## 3. Results and Discussion

### 3.1. Structural Characterization of Au NPs and Au/C NPs

**Figure 1:**
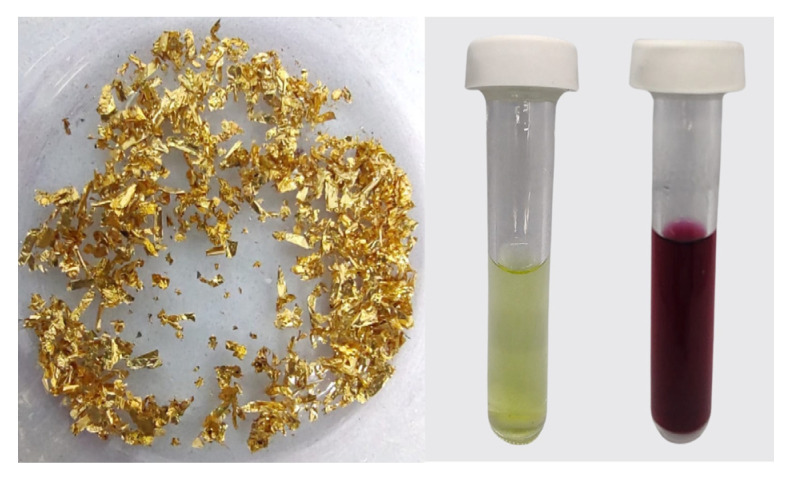
From left to right: gold coatings, HAuCl_4_ (yellowish solution), and unsupported Au NPs (reddish solution) recovered from e-waste.

Figure 1 shows a sequence of processes crucial to our study. First, on the left, are gold coatings meticulously recovered from INTEL^®^ PENTIUM^®^ 4 processors, representing a successful recovery of electronic waste. Moving to the center, we observe the synthesis of HAuCl_4_, which results in a distinctive yellow solution. This synthesis is a fundamental step, involving the transformation of the recovered gold coatings. Finally, on the right, we witness the culmination of the proposed synthesis method: unsupported Au NPs are successfully synthesized from HAuCl_4_, manifesting as a vibrant red-colored solution. This phenomenon arises from Localized Surface Plasmon Resonance (LSPR), a distinctive feature of gold nanoparticles which occurs when the frequency of light coincides with the oscillation frequency of electrons in the nanoparticles [32,33].

This process demonstrates a sustainable approach to harness gold nanoparticles from electronic waste, outlining a new avenue for recycling and electrocatalyst production.

**Figure 2:**
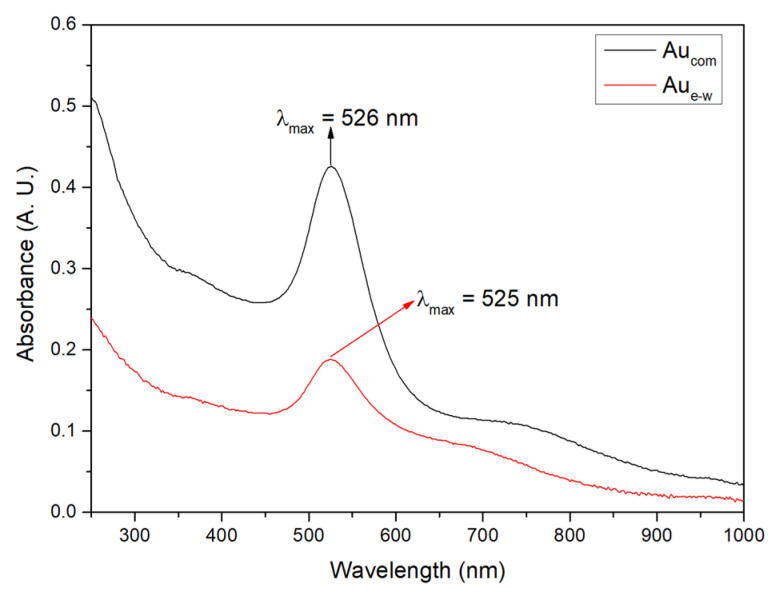
Absorption spectra of the unsupported Au NPs.

Figure 2 shows the UV-Vis spectra of the unsupported Au NPs, with a resonance peak at 526 nm for commercially synthesized gold nanoparticles (Au_com_ NPs) and at 525 nm for those derived from electronic waste (Au_e-w_ NPs). This prominent peak is found to be associated with the Localized Surface Plasmon Resonance (LSPR), indicative of estimated particle sizes within the range of 15 to 20 nm [34]. This observation is in agreement with subsequent TEM analysis, which confirms the consistency of particle size. The slightly higher absorbance intensity observed for Au_com_ NPs compared to Au_e-w_ NPs is noteworthy, pointing to a potentially high concentration of Au_com_ NPs. This speculation is corroborated by subsequent EDS analysis, which further highlights the tonality differences between the two sources.

**Figure 3:**
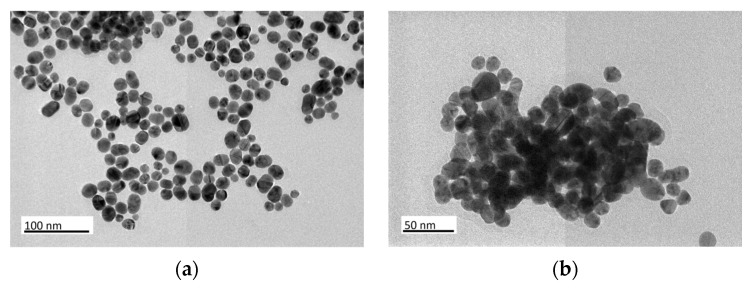

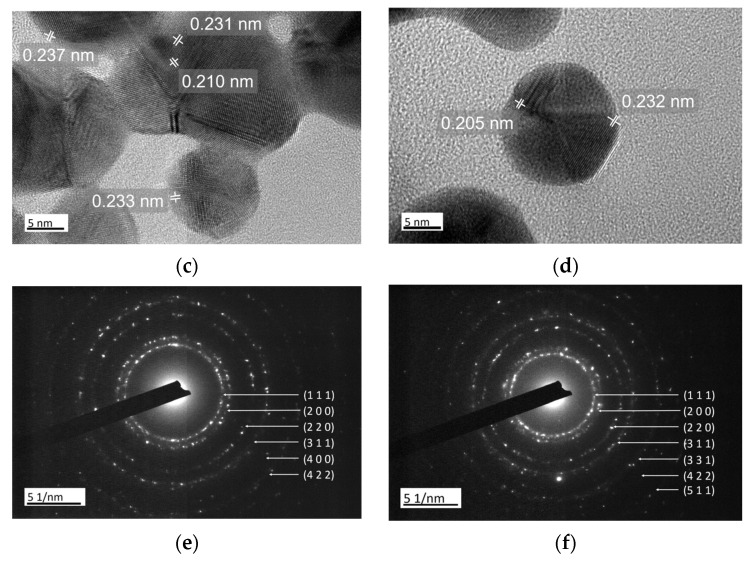
TEM and HR-TEM micrographs, and SAED patterns of the unsupported Au NPs (**a**,**c**,**e**) for Au_e-w_ and (**b**,**d**,**f**) for Au_com_, respectively.

TEM micrographs reveal that the unsupported Au_e-w_ NPs (Figure 3a) predominantly exhibit a spherical morphology with an average particle size of 20.8 ± 5.1 nm. Similarly, the unsupported Au_com_ NPs appear spherical (Figure 3b), with a slightly smaller average particle size of 18.5 ± 2.7 nm. In addition to the spherical particles, both samples contain oblate particles. Moreover, unsupported Au_e-w_ NPs demonstrate a broader size distribution compared to unsupported Au_com_ NPs.

HR-TEM enables the estimation of the crystalline structure of the unsupported Au NPs. In this way, Figure 3c shows that the interplanar distances of unsupported Au_e-w_ NPs are approximately 0.235 nm (with values ranging from 0.231 to 0.237 nm) and 0.210 nm. Similar interplanar distances of 0.232 nm and 0.205 nm are observed for unsupported Au_com_ NPs (Figure 3d). These interplanar distances correspond to the (1 1 1) and (2 0 0) atomic planes of the face-centered cubic (FCC) structure of gold, respectively.

To confirm the presence of the FCC structure of the unsupported Au NPs, SAED experiments were conducted. The SAED pattern of the unsupported Au_e-w_ NPs (Figure 3e) reveals distinct planes, including (1 1 1), (2 0 0), (2 2 0), (3 1 1), (4 0 0), and (4 2 2). Similarly, the SAED pattern of the unsupported Au_com_ NPs (Figure 3f) exhibits planes such as (1 1 1), (2 0 0), (2 2 0), (3 1 1), (3 3 1), (4 2 2), and (5 1 1). All of these atomic planes are consistent with the FCC structure of unsupported Au NPs, as confirmed by the crystallographic sheet PDF 01-1172.

**Figure 4:**
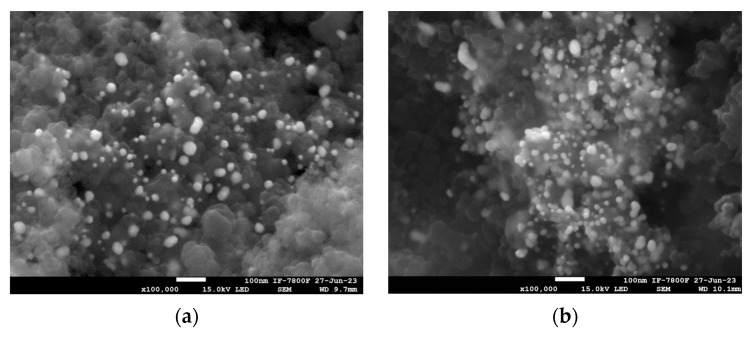

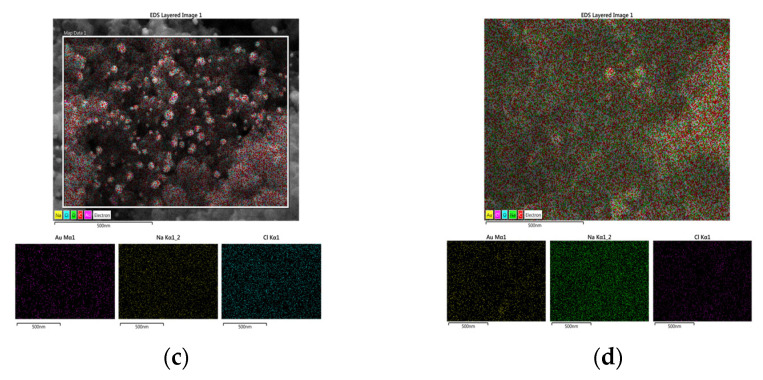
SEM micrographs and SEM color mapping of the supported Au NPs. (**a**) and (**c**) for Au_e-w_/C NPs; (**b**) and (**d**) for Au_com_/C NPs, respectively.

On the other hand, Figure 4 shows the SEM micrographs and the SEM color mapping of the supported Au NPs, where a uniform distribution of gold nanoparticles on Vulcan is observed for both Au_e-w_/C NPs (Figure 4a,c) and Au_com_/C NPs (Figure 4b,d). EDS analysis of Au/C NPs, as summarized in Table 1, shows the presence of elements such as Au, Na, Cl, and O in both samples. The weight percentage of Au is approximately 1.7 times lower in Au_e-w_/C NPs compared to Au_com_/C NPs. These weight percentages of Au are close to the estimated loadings (~20 wt% Au/C NPs). On the other hand, the presence of Na and Cl is due to the NaCl by-product of the synthesis of Au NPs, which, according to the literature, is an expected by-product for this kind of synthesis [35]. Thus, it would be necessary to improve the recovery method of the materials (Au/C NPs) to reduce or eliminate the presence of this by-product.

**Figure 5:**
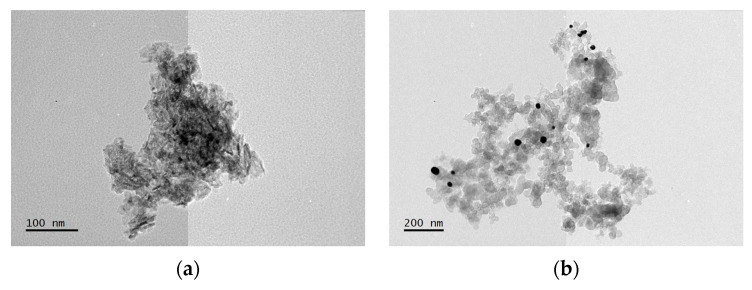

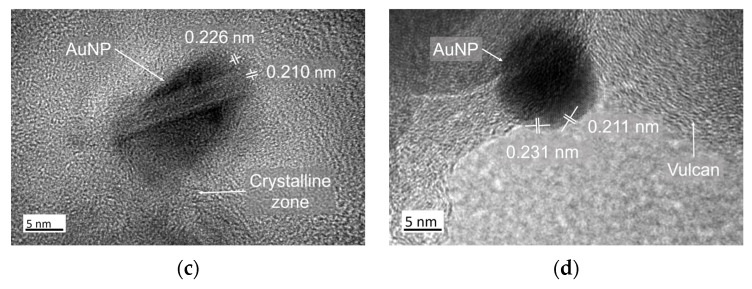
TEM and HR-TEM micrographs of the supported Au NPs. (**a**) and (**c**) for Au_e-w_/C NPs; (**b**) and (**d**) for Au_com_/C NPs, respectively.

TEM micrographs confirm that Au_e-w_ (Figure 5a) and Au_com_ NPs (Figure 5b) are uniformly dispersed on Vulcan and do not form agglomerates. The Au_e-w_/C NPs exhibit a particle size of 20.8 ± 5.1 nm, while the Au_com_/C NPs have a particle size of 18.5 ± 2.7 nm. Notably, the Au_e-w_/C NPs (Figure 5a) show a more crystalline phase on the Vulcan surface. These crystalline areas are likely associated with NaCl (a by-product of the synthesis process). HR-TEM confirms the crystalline structure of the Au NPs supported on Vulcan, revealing interplanar distances of 0.226 nm and 0.210 nm for Au_e-w_/C NPs (Figure 5c), and 0.231 nm and 0.211 nm for Au_com_/C NPs (Figure 5d). These distances are assigned in both cases to the (1 1 1) and (2 0 0) planes. HR-TEM studies also reveal the presence of crystalline regions in the Au_e-w_/C NPs (Figure 5a) that do not belong to either Au NPs or Vulcan (C), which may be attributed to the presence of NaCl. Vulcan exhibits atomic planes characteristic of an amorphous material (Figure 5d).

**Figure 6:**
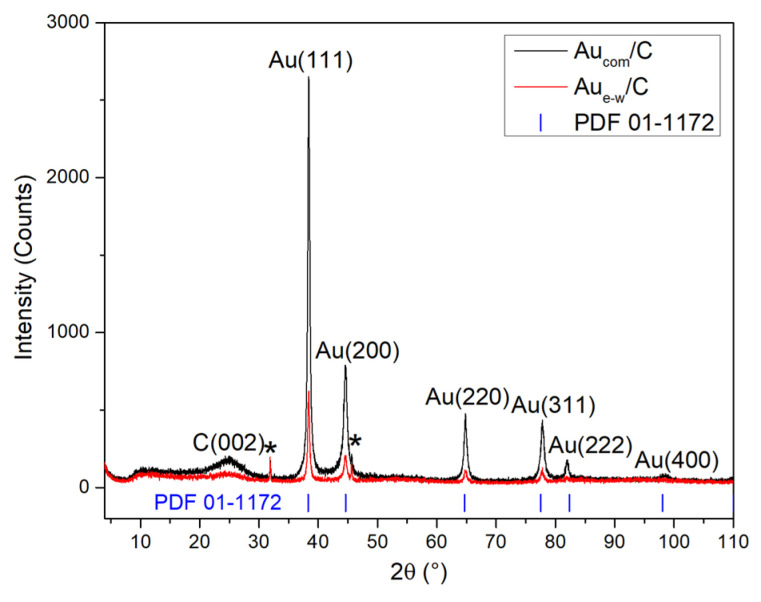
XRD patterns of the supported Au NPs.

Powder XRD patterns (Figure 6) of the supported Au NPs show reflections at 24.97°, 31.85°, 38.41°, 44.54°, 45.60°, 64.78°, 77.78°, 81.90°, and 98.51° for Au_e-w_/C NPs; and 25.02°, 31.87°, 38.39°, 44.54°, 45.60°, 64.84°, 77.80°, 81.96°, and 98.51° for Au_com_/C NPs. In both patterns, the reflections situated at ~38.4°, 44.5°, 64.8°, 77.8°, 81.9°, and 98.5° correspond to the (111), (200), (220), (311), (222), and (400) planes of the Au-FCC structure, confirming the results obtained in SAED experiments (Figure 3e,f). The broadened reflection at ~25° corresponds to the (002) carbon planes of the Vulcan. In addition, reflections at ~31.9° * and ~44.5° * correspond to the (200) and (220) planes, respectively, of sodium chloride, according to ICSD crystallographic record number 96-900-6377. These reflections corroborate the presence of NaCl in the materials, indicating its appearance as a by-product during the synthesis of Au NPs by the method described above. It is also observed that the XRD patterns are slightly more intense for the Au_com_/C NPs, which could be associated with a higher amount (wt%) of Au in this material, as observed by UV-VIS spectroscopy and corroborated by EDS studies.

These results support the crystalline quality of the Au NP, as well as providing additional information on the presence of NaCl as a by-product, as well as a lower gold content (wt%) for the Au_e-w_ NPs.

### 3.2. Electrochemical Characterization of Au/C NPs

**Figure 7:**
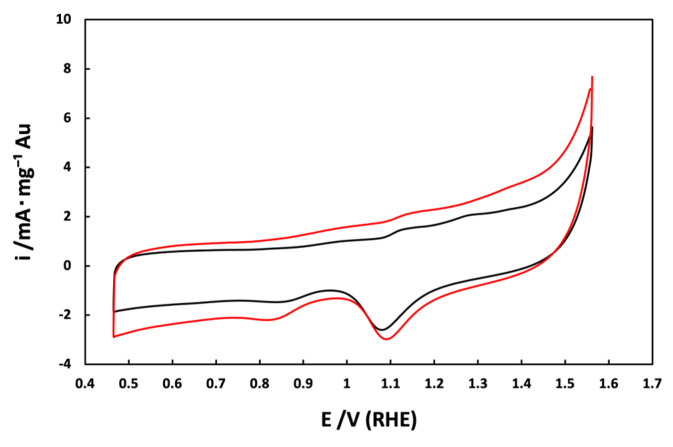
Cyclic voltammograms of the supported Au NPs: (**—**) Au_e-w_/C NPs and (**—**) Au_com_/C NPs in 0.5 mol L^−1^ KOH as electrolyte and at a sweep rate of 20 mV·s^−1^.

Figure 7 shows the cyclic voltammograms in the absence of methanol for Au_e-w_/C NPs (red line) and Au_com_/C NPs (black line). The cyclic voltammograms are normalized with respect to mass current density (mA·mg^−1^_Au_). For both materials, distinct regions characteristic of Au NPs in alkaline media can be identified [29,36]: from 0.93 to 1.06 V/RHE, the anodic region of OH^-^ adsorption and pre-oxidation species formation on the Au NPs surface are observed; from 1.06 to 1.21 V/RHE, processes of superficial oxidation of Au NPs occur; from 1.21 to 1.4 V/RHE, the gold oxide monolayer is formed; from 1.25 to 0.98 V/RHE, a cathodic peak associated with the reduction of surface oxides of Au NPs is observed; and from 0.98 to 0.75 V/RHE, a cathodic peak associated with OH^−^ desorption is also observed. The only difference between the two materials is that Au_e-w_/C NPs show a slightly higher current intensity than Au_com_/C NPs, mainly in the oxygen evolution zone (>1.5 V/RHE).

**Figure 8:**
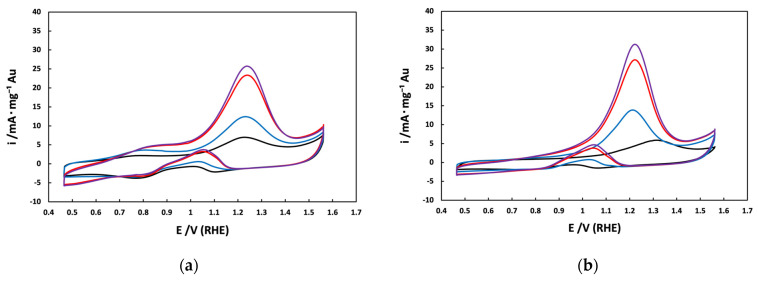
Cyclic voltammograms of the supported Au NPs in the presence of methanol: (**—**) 1 M, (**—**) 2 M, (**—**) 4 M, and (**—**) 5 M in 0.5 mol L^−1^ KOH as electrolyte and at a sweep rate of 20 mV·s^−1^. (**a**) Au_e-w_/C NPS; (**b**) Au_com_/C NPs.

Figure 8 illustrates the cyclic voltammograms recorded in the presence of methanol (ranging from 1 to 5 M) for Au_e-w_/C and Au_com_/C NPs. In the forward scan, it is evident that for Au_com_/C NPs (Figure 8b), only one anodic peak (at 1.22 V/RHE) for methanol oxidation is observed, while for Au_e-w_/C NPs (Figure 8a), two anodic peaks (at 0.80 and 1.22 V/RHE) for methanol oxidation are detected. Previous literature suggests that intermediates/products of the methanol oxidation reaction, such as formaldehyde, formate, and CO, may undergo further oxidation [28,37]. Therefore, it is speculated that a slight amount of intermediates is found accumulating locally, leading to the unexpected first anodic peak observed at 0.80 V/RHE. Additionally, as the methanol concentration increases, the peak value (at 1.22 V/RHE) of the anodic mass-specific current density significantly enhances with the rising methanol concentration.

It is also worth noting that the mass-specific current density for Au/C NPs derived from e-waste is just slightly lower than that obtained from the commercial reagent. For example, at a methanol concentration of 5 M, they exhibit values of 25.5 and 30.1 mA·mg^−1^_Au_, respectively. Nevertheless, the observed activity of Au nanoparticles in the methanol oxidation reaction remains comparable between the two sources. It is also important to note that these values are in agreement with those documented in the existing literature [29,36].

**Figure 9:**
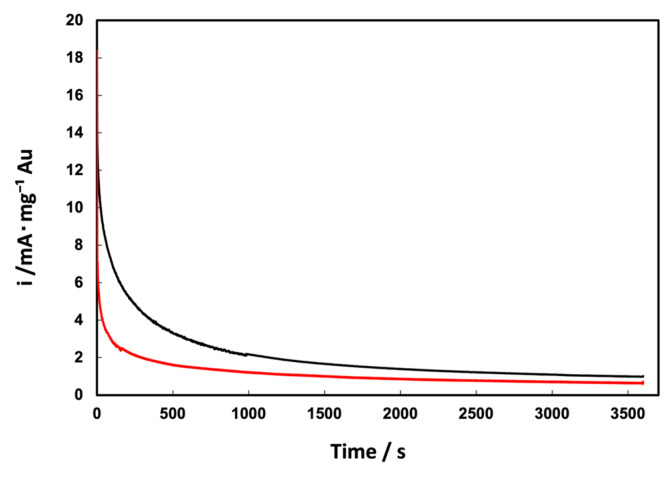
Chronoamperograms in 5 M methanol of the supported Au NPs: (**—**) Au_e-w_/C NPs and (**—**) Au_com_/C NPs in 0.5 mol L^−1^ KOH as electrolyte at 1.17 V/RHE.

Finally, the long-term stability of the supported Au NPs was evaluated by chronoamperometric tests in 5 M methanol (Figure 9) at 1.17 V/RHE to avoid the formation of the gold oxide monolayer, which could affect the methanol oxidation reaction [29]. In general, Au_com_/C NPs show slightly better stability than Au_e-w_/C NPs, particularly at shorter times (<500 s), during which Au_e-w_/C NPs show a more pronounced decrease in current density. However, at longer times (>1500 s), the current density remains relatively constant for Au_e-w_/C NPs, while it continues to decrease for Au_com_/C NPs. After 3600 s of chronoamperometric testing, the difference in current densities between the two materials is minimal.

These electrochemical studies suggest that Au/C NPs obtained from electronic waste show a potential application as an anode in alkaline fuel cells, i.e., for the methanol oxidation reaction.

## 4. Conclusions

The research presented delved into the electrocatalytic potential of Vulcan-supported gold nanoparticles from electronic waste, specifically Intel Pentium 4 processor pins. By synthesizing gold nanoparticles from electronic waste, the possibility of using electronic waste as electrocatalyst precursors was highlighted.

The most remarkable aspect of the presented study is the demonstration that the electrocatalytic activity of gold nanoparticles recovered from e-waste recycling (25.5 mA·mg^−1^_Au_) is similar to that of their commercially synthesized counterparts (30.1 mA·mg^−1^_Au_), affirming the feasibility of this novel approach for the sustainable production of electrocatalysts.

In essence, the research demonstrates the possibility of using electronic waste as a valuable source of electrocatalytic materials. This approach not only addresses environmental concerns related to e-waste, but also offers a practical solution to technological challenges in the field of electrocatalysts for alkaline fuel cells.

## Figures and Tables

**Table 1:** Chemical composition by EDS analysis of the supported Au NPs.

Element	wt% (Au_e-w_/C NPs)	wt% (Au_com_/C NPs)
Au	16.40	27.29
Na	31.84	19.95
Cl	43.55	18.12
O	8.21	34.64

## Data Availability

The data presented in this study are available on request from the corresponding author due to [ethical reasons].
